# Catch me if you can! How French adolescents seize social occasions and opportunities to be active

**DOI:** 10.1186/s12889-022-13746-0

**Published:** 2022-07-12

**Authors:** Thibaut Derigny, Christophe Schnitzler, Teun Remmers, Dave Van Kann, Joseph Gandrieau, Ndongo Seye, Georges Baquet, François Potdevin

**Affiliations:** 1 University Lille, University Artois, University Littoral Côte d’Opale, ULR 7369 - URePSSS - Unité de Recherche Pluridisciplinaire Sport Santé Société, F-59000 Lille, France; 2 Sport et Sciences Sociales, E3S, UR1342, University of Strasbourg, Strasbourg, France; 3 School of Sport Studies, Fontys University of Applied Sciences, Eindhoven, 5644 HZ The Netherlands; 4 Department of Health Promotion, Maastricht University (Medical Center+), NUTRIM School of Nutrition and Translational Research in Metabolism, Maastricht, the Netherlands; 5 Faculty of Mathematiques and applications, speciality Statistiques, University of Strasbourg, Strasbourg, France

**Keywords:** Physical activity, Health, Temporal patterns, Social times, Contexts, Logbook, Accelerometers

## Abstract

**Background:**

Following an ecological framework, the aim of this study was to highlight the way adolescents invested their time in opportunities to engage in moderate to vigorous physical activity (MVPA) according to whether they were profiled as more or less active. This study’s innovation lies in the analysis of MVPA according to social occasions which are understood as opportunities to be active throughout the day (e.g. home, school, transport).

**Methods:**

PA data measured by accelerometry (ActiGraph GT3X) for seven consecutive days were compiled, with adolescents’ social occasions during the week recorded in a daily digital diary (*n* = 135). The opportunity ratio of MVPA at each social time is the ratio between time spent in MVPA and the duration of a corresponding social occasion. Following the literature, participants were categorised into three profiles according to their reported amount of MVPA: HEPA active, minimally active and inactive. Non-parametric Wilcoxon signed rank and Kruskal Wallis tests were performed to determine the relative intensity of PA performed at different social occasions, and to investigate whether intensities differed between adolescents with various activity profiles.

**Results:**

Results showed that engagement in MVPA at different social occasions differed according to participant profiles. Mismatch was noticed between the opportunity ratio and the duration of the most and least favorable social occasions for MVPA. For all three profiles, the social occasion “physical education lesson” revealed an opportunity ratio of MVPA (23.6% vs 17.0% vs 13.8%) significantly higher than the overall opportunity ratio of the week (6.9% vs 2.9% vs 1.2%), but of lower duration. Conversely, “home” (5.3% vs 0.0% vs 0.0%) and “school” (outside of PE time) (2.4% vs 0.0% vs 0.0%) represented the two least opportune social occasions for PA in an adolescent’s week.

**Conclusions:**

Rethinking engagement with MVPA in the context of temporal opportunities would allow potential ways to intervene within an educational supervised setting to help young people adopt a physically active lifestyle at the end of the key period of adolescence. These results reinforced the importance of context in interventions for PA promotion, opening for “time education” in people.

## Background

Despite growing evidence of positive relationships between physical activity (PA) and health, a consensus has emerged on insufficient levels of PA in adolescents [1]. Physical activity, traditionally defined as “any bodily movement produced by skeletal muscles that results in energy expenditure” [2], is considered the cornerstone for maintaining and developing healthy lifestyle habits in adolescents [3]. Recently, a new conception has proposed PA as “people moving, acting and performing within culturally specific spaces and contexts, and influenced by a unique set of interests, emotions, ideas, instructions and relationships” [4]. This epistemological shift moves beyond the uniqueness of biomedical and energetic perspectives to investigate the relationships between environmental affordances (invitations for behaviour) and context-specific PA.

Moderate to vigorous PA (MVPA) is a minor type of activity during the day, yet its health benefits are widely documented [5, 6]. Adolescence is a period of many opportunities for practising MVPA [7, 8], but barriers have been noted in the literature, with the most reported being ‘lack of time’ [9, 10]. Epidemiological studies have considered MVPA as a chronological process, showing that it is not evenly distributed throughout the day [11, 12]. In other words, there are different social temporal periods that structure daily life favorable for undertaking MVPA [13], suggesting that identifying them might be key to improve overall levels of PA.

This approach is framed theoretically by an ecological model [14], involving a complex and holistic perspective on human behavior emerging from contextual interaction with five determinants to engage in MVPA, ranging from individual to macro (environmental) systems [15, 16]. The chronosystem has been conceptualized as including these five systems, following an evolution of pre-defined sequences throughout life, but without investigating temporal opportunities [14]. Several studies have focused on an individual’s ecological determinants, showing that adolescents invested MVPA differently, depending on their activity level profile [17, 18], body mass index (BMI) [19] and gender [20]. However, the holistic structure of the model requires the need to consider the relationship between individual, environmental, and chronological determinants, adopting a perception of time [21] which views specific occasions as providing opportunities (affordances) for engaging in MVPA.

This study addressed the following research question: Which social occasions were the most opportune for adolescents to engage in MVPA, in relative and absolute terms? Through use of objective (accelerometers) and subjective (daily logbooks) measures, the aim of this study was to record the distribution of MVPA undertaken at different social occasions. We hypothesized that total time, as well as MVPA (relative) opportunities taken on different social occasions, would differ according to the adolescents’ PA profile.

## Methods

### Study design and participants

This research recruited a volunteer cohort of adolescents (*n* = 119 after removals – 135 before; age_mean_ = 17.03 ± 0.7 years old; 74 girls and 45 boys) from five secondary schools (Strasbourg and Lille, France). Before entering into the study, written consent was obtained from the adolescents and parents/caregivers, if they were under 18 years old. Data collection occurred between October 10th and November 15th, 2020. This period was impacted by the second French lockdown where lifestyles were adapted following school closures [22].

Schools were randomly selected based on the level of urbanization of their location (varying from rural to urban). In each school, a second randomization has been carried out to select two classes. Gender and BMI parities criteria were investigated to not impact the initial distribution of the physical activity observed [19, 20]. Inclusion criteria were to be in the last year of secondary school in France, to agree to wear an accelerometer for 1 week and to complete a daily diary for 7 days. For all recruited participants, we collected sociodemographic data including age, sex, height, weight, home and e-mail address and telephone number. This study was conducted according to the guidelines of the Declaration of Helsinki [23] and approved by the Ethics Committee of the University of Lille (2020-418-S82) and the NCIF (National Committee for Informatics and Freedoms number 2020-037, approval May 2020).

### Outcome measures

ActiGraph accelerometers, model GT3X+ (ActiGraphTM, Pensacola, FL, USA), were used to measure PA with a sampling rate of 30 Hz. Participants wore the accelerometer on their preferential hip, fastened with an elastic belt for 7 full days [24, 25]. Data were reintegrated using a 10-s epoch. Troiano’s (2007) wear time validation algorithm [26] was applied, associating non-wear time to all periods > 60 minutes of consecutive counting at zero. The Actigraph output of 1952 counts.min^− 1^ was the cut-offs used to define the intensity level of MVPA [27].

During the week of data collection, participants completed a digital diary on the LimeSurvey platform to obtain information about their daily social activities. Participants completed the diary every night before going to bed, which took about 5 min. Questionnaires were based on pre-existing studies including twelve typical social occasions through daily diaries [11, 13]: (a) autonomous leisure, (b) recess, (c) cleaning, (d) home, (e) homework, (f) job, (g) meal, (h) PE lesson, (i) relax, (j) school, (k) supervised leisure and (l) transport. For clarity, autonomous leisure activities are all social activities which can be done alone or in a group (e.g. shopping or jogging alone or with others). In contrast, supervised leisure times included PA like sport club, but also all supervised leisure such as music and drama classes, supervised by a specialist of this activity. The PE lesson occasion is included in school time but required a specific attendance. Some other social occasions may be subject to temporal intersections, such as those at school with PE lesson and recreation (breaks between lessons), or those at home with cleaning, rest, homework and meals. The priority category was systematically given to the most precise (e.g. recreation priority over school, or rest over home). Finally, transport corresponds to all modes of travel used, whether active (walking, electric scooter or bicycle) or inactive (cars, bus, subway).

### Data treatment

**Fig. 1:**
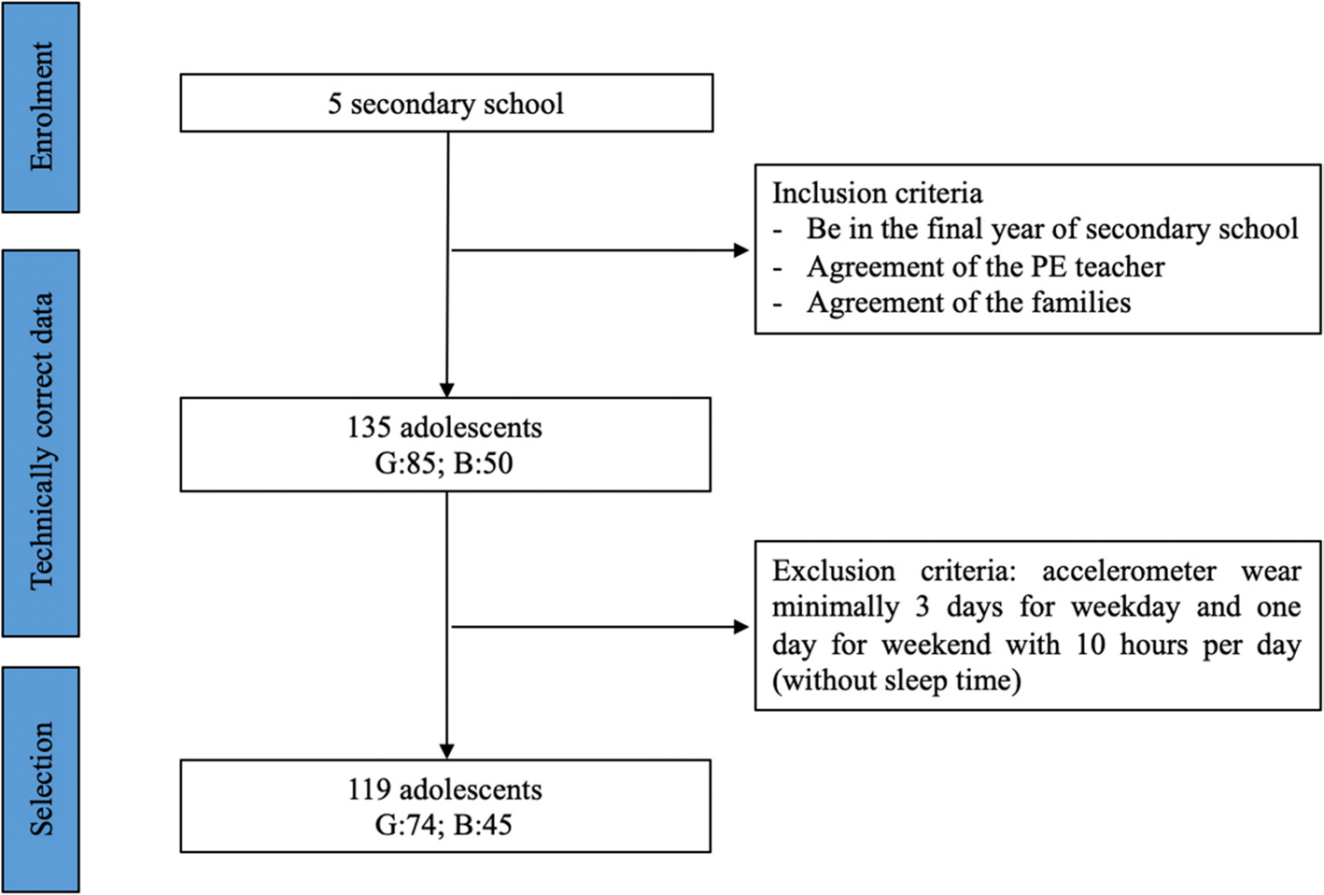
Flowchart of selection of participants (G:girls; B:boys)

The flowchart is presented in Fig. 1. A minimum of three weekdays and one weekend day, with valid PA data (≥10 h per day), was required to be included in the analyses [24, 28]. Sleep time was excluded. Data were excluded when adolescents experienced unexpected drastic changes in their lifestyle during the data collection (e.g. periods of sickness). According to the time spent in MVPA per day, collected by accelerometers, participants were categorized into a PA profile [17, 18]: Health Enhancing Physical Activity (HEPA) active adolescents spent at least 60 minutes in MVPA per day, minimally active adolescents spent between 30 and 60 minutes and inactive adolescents spent less than 30 minutes in MVPA per day.

### Statistical analysis

Statistical analyses were conducted with R software (version 4.1.0), with specific packages (tidyverse, outliers, psych, car, rstatix, ez, lsmeans, pwr, Rmisc, ggplot2). Data and residuals of age, BMI, time spent in sedentary behavior, low physical activity and MVPA for each profile (HEPA active, minimally active and inactive) were tested for normality, interdependence and homoscedasticity using Shapiro-Wilks, Levene and Jarque-Bera tests. As these preliminary conditions were not met, non-parametric tests on median and quartile values were used. The threshold of statistical significance was set at 5% (*p* < .05).

Chi-squared and Kruskal-Wallis tests were used in order to detect profile effects on gender, age, BMI, time spent in each PA categories and time spent in each social occasion. We converted wear times of accelerometers proportionally for a comparative duration of 7 days. We expressed time spent in each social occasion in minutes per week.

The second step of analysis consisted of contrasting, for the three profiles, the duration of each social occasion with its MVPA opportunity ratio (%MVPA). Opportunity ratio values were calculated by dividing the time spent in MVPA by the duration of the same social occasion. For each profile, one sample Wilcoxon signed rank test was used to compare the opportunity ratio of MVPA of each social occasion with the median opportunity ratio of the week. In order to detect significant effects of PA profiles on each duration and opportunity ratio of MVPA, Kruskal-Wallis tests were performed. We used pairwise Wilcoxon tests with Holm correction as a post hoc test to highlight differences between each profile. Last, we calculated Partial Eta squared values (η_p_^2^) and their confidence intervals, to examine effect sizes, considered as small when η_p_^2^ > .01, medium when η_p_^2^ > .06, and large when η_p_^2^ > .14 [29].

## Results

### Profile of the participants

Characteristics of the study sample according to each PA profile are presented in Table 1. The three groups were homogeneous according to gender, age and BMI characteristics. Accelerometer data highlighted that the distribution of percentages of time spent in MVPA (χ ^2^(2) = 103.97, *p* < .05, η^2^_P_ = .88, large) were significantly different across the three profiles.

**Table 1:** Descriptive characteristics of study samples according to physical activity profiles

	HEPA active	Minimally active	Inactive	Profile effect		
				*p*-value	η^2^_P_	χ^2^
N	47	32	40	–	–	–
Gender						
Girls	33	21	20	NS	–	–
Boys	14	11	20			
Age (years) ^#^	16.96 ± 0.55	16.97 ± 0.97	17.18 ± 0.59	NS	.01 [.01; .11]	(2) =3.33
BMI (kg/m^2^) ^#^	21.78 ± 2.85	21.4 ± 2.87	21.22 ± 3.26	NS	.01 [.01; .08]	(2) =1.20
Time spent in MVPA (min, %) ^^^	443 [319; 521] (6.9%)	364 [250; 339] (2.8%)	120 [72; 171] (1.2%)	<.05 ^abc^	.88 [.85; .89]	(2) =103.97

^#^: data are in mean ± standard deviation; ^^^: data are in median of minutes per week [Q1; Q3] and % of time spent in MVPA as a function of total accelerometer-wearing time; *NS* Non-Significant, *HEPA* Health Enhancing Physical Activity, *BMI* Body Mass Index, MVPA Moderate-to-Vigorous Physical Activity. ^a^: Statistical significant difference on percentage of time in MVPA between HEPA active and minimally active; ^b^: Statistical significant difference in percentage of time in MVPA between HEPA active and inactive; ^c^: Statistical significant difference in percentage of time in MVPA between minimally active and inactive

### Opportunity ratio of MVPA compared to duration of each social time

Data on social occasion durations for each profile and their corresponding opportunity ratio (%MVPA) are presented in Table 2. The PE lesson was the only social occasion with an opportunity ratio of MVPA significantly higher than the overall opportunity ratio for all profiles (23.6, 17.0, 13.8%, vs 6.9, 2.9, 1.2% for HEPA active, minimally active and inactive profiles respectively). For the HEPA active profile, the two other social occasions with a significantly higher opportunity ratio were recess (12.9%) and transport (9.4%). All other social times had a significantly lower opportunity ratio (*p* < .05), except autonomous leisure time (6.1% with *p* > .05 which was not statistically significant). Regarding minimally active and inactive profiles, autonomous leisure time was the only other social occasion that had a significantly higher MVPA opportunity ratio than the overall median (3.4 and 3.6% vs 2.9 and 1.2% respectively, *p* < .05). All other social times had a significantly lower score than the overall opportunity ratio (*p* < .05), except for recess and transportation (lower, but not statistically significant).

**Table 2:** Opportunity ratios of MVPA and durations for each social time according to adolescent’s profile

Social times		HEPA active	Minimally active	Inactive	Profile effect		
					*p*-value	η_p_^2^	χ^2^
Overall	%MVPA	6.9% [5.2; 8.3]	2.9% [2.5; 3.4]	1.2% [1; 2]	<.05 ^abc^	.89 [.85; .88]	(2) =103.96
Autonomous Leisure	%MVPA	6.1% [4.6; 44]	3.4% [0.6; 7.2] *	3.6% [0; 4.8] *	NS	.01 [.01; .11]	(2) =3.3153
	Duration	60 [0; 893]	74 [0; 680]	97 [0; 1305]	<.05 ^ab^	.18 [.06; .35]	(2) =22.409
Cleaning	%MVPA	0.0% [0; 0.3] *	0.0% [0; 0] *	0.0% [0; 0] *	NS	.09 [.03; .18]	(2) =5.8309
	Duration	0 [0; 13]	9 [0; 70]	3 [0; 119]	<.05 ^b^	.06 [.01; .02]	(2) =8.8436
Home	%MVPA	5.3% [3.4; 7.6] *	0.0% [0; 2.5] *	0.0% [0; 1.6] *	<.05 ^ab^	.37 [.21; .54]	(2) =44.441
	Duration	2056 [951; 3339]	2198 [1168; 5879]	1889 [485; 4515]	<.05 ^b^	.06 [.01; .19]	(2) =8.7997
Homework	%MVPA	0.0% [0; 0.7] *	0.0% [0; 0] *	0.0% [0; 0] *	NS	.02 [.01; .13]	(2) =4.0439
	Duration	82 [0; 203]	77 [0; 656]	102 [50; 718]	NS	.01 [.01; .07]	(2) = .98648
Job	%MVPA	0.0% [0; 0] *	0.0% [0; 0] *	0.0% [0; 0] *	NS	.09 [.03; .20]	(2) =11.972
	Duration	0 [0; 0]	0 [0; 0]	0 [0; 0]	<.05 ^b^	.08 [.03; .18]	(2) =11.115
Meal	%MVPA	0.0% [0; 4.4] *	0.0% [0; 6.6]	0.0% [0; 6]	NS	.18 [.06; .33]	(2) =22.261
	Duration	325 [188; 403]	121 [0; 235]	111 [0; 171]	<.05 ^ab^	.20, [.08; .36]	(2) =25.566
PE lesson	%MVPA	23.6% [12.7; 30.2] *	17% [0; 22.4] *	13.8% [7.2; 18.3] *	<.05 ^ab^	.04 [.01; .14]	(2) =6.8914
	Duration	95 [68; 109]	34 [0; 93]	96 [34; 115]	<.05 ^ab^	.16 [.05; .33]	(2) =20.552
Recess	%MVPA	12.9% [7.6; 16.7] *	0.0% [0; 5.2]	0.0% [0; 4.2]	<.05 ^ab^	.27 [.13; .45]	(2) =32.914
	Duration	76 [52; 105]	30 [0; 64]	60 [30; 165]	<.05 ^ab^	.17 [.06; .34]	(2) =21.8
Relax	%MVPA	3.4% [0; 5.2] *	0.0% [0; 0] *	0.0% [0; 0] *	<.05 ^ab^	.30 [.15; .48]	(2) =36.686
	Duration	595 [0; 1450]	212 [0; 2161]	193 [0; 1915]	<.05 ^ab^	.23 [.09; .41]	(2) =28.853
School	%MVPA	2.4% [1.4; 3.9] *	0.0% [0; 1.8] *	0.0% [0; 1.6]	<.05 ^ab^	.25 [.13; .43]	(2) =31.327
	Duration	1252 [785; 2048]	1187 [581; 2666]	1002 [478; 2569]	<.05 ^ab^	.13 [.03; .29]	(2) =17.14
Supervised Leisure	%MVPA	2.8% [0; 28.9] *	0.0% [0; 0] *	0.0% [0; 0] *	<.05 ^ab^	.19 [.08; .32]	(2) =23.624
	Duration	89 [0; 180]	37 [0; 308]	9 [0; 315]	<.05 ^ab^	.22 [.10; .39]	(2) =27.761
Transport	%MVPA	9.4% [6.1; 15.1] *	0.0% [0; 10]	0.0% [0; 9]	<.05 ^ab^	.14 [.04; .29]	(2) =17.636
	Duration	263 [158; 492]	197 [0; 1278]	127 [72; 545]	<.05 ^ab^	.23 [.10; .41]	(2) =29.045
Other ^	%MVPA	7.6% [5.3; 9.7] *	2.5% [1.6; 3.1]	0.8% [0.3; 1.1]	<.05 ^abc^	.68 [.53; .79]	(2) =81.134
	Duration	5268 [4739; 5997]	5635 [4935; 6124]	6360 [5789; 6743]	<.05 ^abc^	.58 [.47; .69]	(2) =69.545

%MVPA: Opportunity ratio of MVPA; %MVPA are in % median [Q1; Q3]; Duration are in median minutes of weartime per week [Q1; Q3] *: difference between the opportunity ratio of each social time and the overall opportunity ratio with *p* < .05; *NS* Non-Significant; Health Enhancing Physical Activity; ^a^: Significant difference on opportunity ratio of MVPA between HEPA active and minimally active; ^b^: Significant difference on opportunity ratio of MVPA between HEPA active and inactive; ^c^: significant difference on opportunity ratio of MVPA between minimally active and inactive; ^^^: other represents all the social times that could not be reconstructed by diaries

Data showed a significant profile effect on all social occasion (*p* < .05) durations, except for homework. Focusing on the MVPA opportunity ratios (%MVPA), there was a significant effect of profile on each social occasion ratio (*p* < .05) except for five of them: autonomous leisure, cleaning, homework, job and meal. Post-hoc analyses showed that HEPA active adolescents had a significantly higher MVPA opportunity ratio, compared to minimally active and inactive profiles on different social occasions: home (respectively 5.3% vs 0 and 0%, *p* < .05), PE lesson (23.6% vs 17% vs 13.8%, *p* < .05), recess (12.9% vs 0 and 0%, *p* < .05), relax (3.4% vs 0 and 0%, *p* < .05), school (2.4% vs 0 and 0%, *p* < .05), supervised leisure (2.8% vs 0 and 0%, *p* < .05), and transport (9.4% vs 0 and 0%, *p* < .05). In contrast, the MVPA opportunity ratios for participants who reported being minimally active and inactive did not differ significantly across any social occasion.

## Discussion

The objective of this study was to examine how French adolescents accumulate MVPA according to their PA profiles, at different social occasions during the day. The three main results were that: (a) the PE lesson provides the best MVPA opportunities in all profiles, (b) school and at home represent the longest times spent in social contexts, but provide low opportunities for MVPA, and (c) participants in the HEPA active profile manage to seize MVPA opportunities at many social occasions compared to participants in the other two profiles.

### The big gap to engage in MVPA: a mismatch between duration and opportunity ratio for PE lesson, school and at home

Results showed that the PE lesson exhibited the highest opportunity ratio values, thus representing the most opportune social occasion to engage all adolescents in MVPA. These data confirmed those reported by Sanz Martin et al. (2021) and Gavarry et al. (2003) who showed that, on the day the PE lesson took place, adolescents engaged in more MVPA [30, 31]. However, its opportunity ratio level, between 23.6% (HEPA active profile) and 14.7 (inactive profile) was rather low and confirmed the systematic review of Burson et al. (2021) showing that only 31% of the PE lesson is consecrated to MVPA [32]. Its duration in the week (95, 34 and 96 minutes per week for each profile) was insufficient to ensure enough weekly MVPA levels [8, 33]. While the literature was rich regarding pedagogical methods to increase adolescents’ MVPA [34], the discrepancy between participants in terms of opportunity ratio values (23.6% vs 13.8%, *p* < .05, for HEPA active and inactive profiles respectively) suggest the need to (a) develop specific teaching strategies according to each PA profile in PE [35], (b) increase PE time per week, and (c) inform parents and adolescents to the actual amount of MVPA during PE classes to ensure they are aware of the need to engage in the MVPA on other social occasions to reach the WHO’ guidelines.

The time reported in PE lessons for the minimally active profile (34 minutes per week) seems low considering that at this age, a minimum duration of PE is set at a standard of 120 minutes per week in the French education system. This difference of engagement, compared to values reported by HEPA active and inactive students, is hard to explain from the data and could perhaps be attributed to the disruptions brought about by the COVID-19 (CV19) pandemic, which interrupted PE lessons in some classes and not others [36]. However, as we were mostly interested in identifying opportune times for MVPA, we believe that this anomaly does not negate our conclusions.

Home (5.3% vs. 0.0% vs. 0.0% for HEPA active, minimally active and inactive profiles respectively) and school (2.4% vs. 0.0% vs. 0.0%) represent the venues for the two social occasions with the significantly lowest opportunity ratio values. Logically, the higher the duration of social occasions, the lower should be the opportunity ratio values, corroborating previous studies showing that school time tends to promote sedentary behaviors during the day [37, 38]. Paradoxically, school has been considered as the most appropriate place to promote a PA lifestyle for adolescents [39–41]. Brooke et al. (2014) have shown that adolescents had a total PA level lower in school, than out of school, with the possibility of accumulating even more in it [42]. Weaver et al. (2021) have shown that MVPA has shown increases during school time since 2015 [38]. Therefore, it seems apparent that the pedagogical challenge consists of tailoring programs to impact on minimally active and inactive adolescents during school time, including PE time in this strategy. Focusing on home social occasion, result highlighted the importance of the time spent at home to accumulate MVPA. Without quality support, adolescents did not engage independently in this social occasion, which was a significant part of their week.

### The “HEPA active” profile: a population model to seize MVPA’ opportunities

One of the main findings of this study was that participants in the HEPA active profile managed to accumulate higher values of MVPA than participants in the two other profiles on many social occasions, including: home, PE lesson, recess, relax, school, transport, autonomous and supervised leisure (*p* < .05 and η_p_^2^ > .14). These social occasions seem important to move from inactive to HEPA active profile. Considered as “a role model group” in terms of MVPA, analysis of participants with the HEPA active profile have shown an ability to optimize social times for undertaking MVPA, compared to participants in other profiles.

Except for PE lesson, the two higher values of the opportunity ratio for HEPA active adolescents were transport and recess social occasions (respectively 12.9 and 9.4%). Level of transport supports the rationale for promoting use of methods of active transportation to tackle the decline in PA in the adolescent population. Booth et al. (2014) have reported a downward trend in active transportation use among adolescents over the past two decades [43]. Conversely, several intervention studies with environments built around cycling to school have shown positive results in terms of increasing MVPA [44, 45]. Focusing on recess time results have reinforced the importance of this scholarly time in high school to promote MVPA, confirming findings in studies focusing exclusively on younger children (primary and secondary schools) with playground facilities [46, 47]. Thus, our results reinforced the need to promote both active recess periods during school and active transportation before and after school.

Autonomous leisure, home and relax social occasions, demonstrated the ability of HEPA active participants to accumulate MVPA on their own during free time, despite displaying a lower ratio than the median value (respectively 6.1, 5.3 and 3.4% vs 6.9%). These results were in line with current French studies that show a shift in PA practice among adolescents towards free activities without constraints [48, 49]. These results corroborate the low opportunity ratios of supervised leisure social occasion (2.8, 0, 0% for each PA profile), perceived as collateral damage of these new unconstrained practices. Specifically in adolescents, previous studies have shown that sports participation was socially stratified, for example regarding school program [50], gender [50–52] or parental PA participation [50]. This study did not consider socioeconomic status and the relationships that parents have with PA but outlined that this supervised leisure time could also provide opportunities to increase MVPA levels.

It is worth noticing participants in the HEPA active profile accumulated MVPA on five social occasions which were totally ignored by participants in other two profiles, while their absolute duration values were almost identical: home (5.3% vs 0% vs 0%), relax (3.4% vs 0% vs 0%), recess (12.3% vs 0%vs 0%), transport (9.4% vs 0% vs 0%) and autonomous leisure (2.8% vs 0% vs 0%). To the best of our knowledge, this result is a novel contribution to the literature, since, so far, studies have mainly analyzed MVPA through the filter of individual determinants of the ecological model (i.e. gender, age, BMI), but rarely by daily social activities [53–55]. This new knowledge reinforces the idea of the importance of education to invest maximum opportunities in a large variety of social occasions, to facilitate individuals to achieve the levels stipulated in the World Health Organization (WHO) guidelines for MVPA [56]. This ability remains one of the most common goals targeted by PE teachers around the world [39]: educate to a physically active, healthy, and sustainable lifestyle [48, 57]. Individuals with a HEPA active profile seem to represent the more physically literate students, and it would be interesting to examine which kind of physical, cognitive, social, and emotional skills and attributes they exhibit [58]. It seems necessary to conduct more longitudinal studies to verify whether they will, in the future, be able to “value and take responsibility for engagement in physical activities for life” [59].

### Creating new social opportunities to fostering MVPA in all adolescents

One other interesting result was the different use of social occasions to accumulate MVPA according to the adolescents’ profiles. The literature has documented consistently that “lack of time” was one of the most frequently reported barriers to engagement in PA [9, 10]. However, our results showed that HEPA active adolescents were able to seize MVPA opportunities in different social occasions, allowing them to reach the recommended PA thresholds. The question of how to promote PA during unsupervised social occasions appears to be key in fostering MVPA in the minimally active and inactive adolescents. It also offers a new perspective for designing PA promotion in an ecological framework [14–16] where the chronosystem would not be considered as continuous, but rather in terms of opportunistic times. Distinctions between the duration and the MVPA opportunity ratio according to the adolescents’ PA profiles demonstrated the need to revisit this ecological model where temporality has, until now, been perceived as linear (chronos) when it would appear to be better considered as in terms of opportunistic moments (kairos). Both educational interventions and territorial disposition for MVPA engagement should be rethought by including “social occasions exploration” to achieve better health outcomes. School curriculum for PA levels among adolescents could be further informed by these findings. It raises the question of adolescents’ exploration of social occasions, opening the way for further qualitative studies to identify conscious and unconscious processes of MVPA engagement during different social occasions.

To the best of our knowledge, little is known about the relationship between time perception and PA opportunities among adolescents [60]. Time has been almost considered as a pre-defined period of chronological segments to depict patterns of PA among adolescents [12, 61, 62], rather than as a perception of opportunities for PA. This vision of social occasions considered as affordances (an “invitation”) to develop PA [63] is needed to better understand PA behaviors in adolescents and tailor strategies to promote PA in this population. The way an individual uses their time can greatly affect their health and results of this study showed the relevance of exploring maximal social times to meet the requisite PA levels stipulated in WHO guidelines. This time question associated with PA has been already framed by the concept of time perspective [60] which represents the personal attitude toward past, present and future times; or the concept of elasticity of time [64] in order to understand ripple effects when time variation in one activity (MVPA) affects the others. Our results encourage future studies to focus deeply on the question of use and perception of time to develop MVPA among adolescents.

### Limits

The strengths of this study are based on the measurement of MVPA by accelerometry and its limitation is in the documentation of social occasions by self-reported questionnaire. The category of “other” referred to all social times when participants were awake, that could not be reconstructed by the logbook. The HEPA active adolescents seemed to be more assiduous than the minimally active and inactive in providing information in their logbooks. Finally, as with all current studies of PA among young people, the context of the CV19 pandemic has had a significant impact on active behaviors. Future studies will need to be conducted to verify whether the trends reported in this study will continue in conditions outside the pandemic.

## Conclusions

Understanding how adolescents organize their social time on different occasions to adopt active and healthy behaviors is a major challenge to better promote and educate people about PA levels. Overcoming a physical barrier of engagement (e.g., constructing a new, attractive and accessible PA affordance) may only lead to an increase in MVPA if there is sufficient environmental support for activity opportunities. Future interventions should incorporate this ecological view of affordances of social occasions to investigate whether engagement in a context may also limit activity opportunities within another. It would be interesting to further develop this temporal analysis of PA with the work of Hägerstand on time-geography to better understand the engagement and mobility in MVPA of adolescents, adopting an ecological perspective on constraints of time and space [65].

## Data Availability

Deidentified individual participant data that underlie the results reported in this article will be made available for 5 years following article publication to researchers who provide methodologically sound proposals. To gain access, data requestors will need to sign a data sharing agreement. Proposals for deidentified data and/or a full trial protocol should be directed to thibaut.derigny@univ-lille.fr.

## Abbreviations

%MVPA: Opportunity ratio of MVPA
BMI: Body Mass Index
COVID-19: Coronavirus Disease 2019
HEPA: Health-Enhancing Physical Activity
MVPA: Moderate Vigorous Physical Activity
η_p_^2^: Partial Eta squared values
PA: Physical Activity
Q1: Quartile 1
Q3: Quartile 3
CV19: COVID-19
WHO: World Health Organization

## References

[1] Guthold R, Stevens GA, Riley LM, Bull FC (2020). Global trends in insufficient physical activity among adolescents: a pooled analysis of 298 population-based surveys with 1·6 million participants. Lancet Child Adolesc Health.

[2] Caspersen CJ, Powell KE, Christenson GM (1985). Physical activity, exercise, and physical fitness: definitions and distinctions for health-related research. Public Health Rep.

[3] Poitras VJ, Gray CE, Borghese MM (2016). Systematic review of the relationships between objectively measured physical activity and health indicators in school-aged children and youth. Appl Physiol Nutr Metab.

[4] Piggin J (2020). What is physical activity? A holistic definition for teachers, researchers and policy makers. Front Sports Act Living.

[5] Carson KV, Chandratilleke MG, Picot J, Brinn MP, Esterman AJ, Smith BJ. Physical training for asthma. Cochrane Database Syst Rev. 2013;9. 10.1002/14651858.CD001116.pub4. 10.1002/14651858.CD001116.pub4 PMC11930393 24085631

[6] Hills AP, Dengel DR, Lubans DR (2015). Supporting public health priorities: recommendations for physical education and physical activity promotion in schools. Prog Cardiovasc Dis.

[7] Guinhouya BC, Lemdani M, Vilhelm C, Hubert H, Apété GK, Durocher A (2009). How school time physical activity is the “big one” for daily activity among schoolchildren: a semi-experimental approach. J Phys Act Health.

[8] Brazendale K, Beets MW, Armonstrong B, Weaver RG, Hunt ET, Pate RR, Brusseau TA, Bohnert AM, Olds T, Tassitano MR, Tenorio MCM, Garcia J, Andersen LB, Davey R, Hallal PC, Jago R, Kolle E, Kriemeler S, Kristensen PL, Kwon S, Puder JJ, Salmon J, Sardinha LB, Van Slujdis EMF (2021). Children’s moderate-to-vigorous physical activity on weekdays versus weekend days: a multi-country analysis. Int J Behav Nutr Phys Act.

[9] Embersin C, Chardon B, Grémy I (2007). Jeunes en Île-de-France : activités physiques, surpoids et conduites à risque. Exploitation du Baromètre Santé 2005. Observation régionale de santé d’Île-de-France.

[10] Duffey K, Barbosa A, Whiting S, Mendes R, Yordi Aguirre I, Tcymbal A, Abu-Omar K, Gelius P, Breda J (2021). Barriers and facilitators of physical activity participation in adolescent girls: a systematic review of systematic reviews. Front Public Health.

[11] De Baere S, Lefevre J, De Martelaer K, Philippaerts R, Seghers J (2015). Temporal patterns of physical activity and sedentary behavior in 10–14 year-old children on weekdays. BMC Public Health.

[12] Wendt A, Wehrmeister FC, Ricardo LIC, Silva BGC, Martins RC, Gonçalves H, Reichert FF, Crochemore-Silva I (2020). Objectively measured physical activity according to the periods of the day in the Pelotas cohort. Revista Brasileira de Atividade Fisica & Saude.

[13] Klinker CD, Schipperijn J, Kerr J, Ersball AK, Troelsen J. Context-specific outdoor time and physical activity among school-children across gender and age: using accelerometers and GPS to advance methods. Front. Public Health. 2014;2. 10.3389/fpubh.2014.00020. 10.3389/fpubh.2014.00020 PMC3949325 24653983

[14] Bronfenbrenner U (1979). The ecology of human development: experiments by nature and design.

[15] Sallis JF, Saelens BE (2000). Assessment of physical activity by self-report: status, limitations, and future directions. Res Q Exerc Sport.

[16] Bauman AE, Reis RS, Sallis JF, Wells JC, Loos RJ, Martin BW (2012). Correlates of physical activity: why are some people physically active and others not?. Lancet.

[17] Derigny T, Schnitzler F, Gandrieau J, Potdevin F (2022). Resilience of adolescents in physical activity during the covid-19 pandemic: a preliminary case study in France. Phys Act Rev.

[18] Von Rosen P, Dohrn IM, Hagstömer M (2020). Lattent profile analysis of physical activity and sedentary behavior with mortality risk: a 15-year follow-up. Scand J Med Sci Sports.

[19] Hemmingsson E, Ekelund U (2007). Is the association between physical activity and body mass index obesity dependent?. Int J Obes.

[20] Slootmaker SM, Schuit AJ, Chinapaw MJ (2009). Disagreement in physical activity assessed by accelerometer and self-report in subgroups of age, gender, education and weight status. Int J Behav Nutr Phys Act.

[21] Élias N (1996). Du temps.

[22] Viner RM, Russell SJ, Croker H (2020). School closure and management practices during coronavirus outbreaks including COVID-19: a rapid systematic review. Lancet Child Adolesc Health.

[23] World Medical Association Declaration of Helsinki (2013). Ethical principles for medical research involving human subjects. JAMA..

[24] Trost SG, McIver KL, Pate RR (2005). Conducting accelerometer-based activity assessments in field-based research. Med Sci Sports Exerc.

[25] Vanhelst J (2019). Quantification de l’activité physique par l’accélérométrie. Rev Epidemiol Sante Publique.

[26] Troiano RP (2007). Large-scale applications of accelerometers: new frontiers and new questions. Med Sci Sports Exerc.

[27] Freedson PS, Melanson E, Sirard J (1998). Calibration of the Computer Science and Applications, Inc. accelerometer. Med Sci Sports Exerc.

[28] Cain KL, Sallis JF, Conway TL, Van Dyck D, Calhoon L (2013). Using accelerometers in youth physical activity studies: a review of methods. J Phys Act Health.

[29] Cohen J (1988). Set correlation and contingency tables. Appl Psychol Meas.

[30] Sanz-Martín D, Ruiz-Tendero G, Fernández-García E (2021). Contribution of physical education classes to daily physical activity levels of adolescents. Phys Act Rev.

[31] Gavarry O, Giacomoni M, Bernard T, Seymat M, Falgairette G (2003). Habitual physical activity in children and adolescents during school and free days. Med Sci Sports Exerc.

[32] Burson SL, Mulhearn SC, Castelli DM, Van Der Mars H (2021). Essential components of physical Education: policy and environment. Res Q Exerc Sport.

[33] Guinhouya BC (2010). Physical activity of schoolchildren in France. The paradox of a public health priority!. Revue d’épidémiologie et de Santé Publique.

[34] Álvarez-Bueno C, Pesce C, Cavero-Redondo I, Sánchez-López M, Garrido-Miguel M, Martínez-Vizcaíno V (2017). Academic achievement and physical activity: a Meta-analysis. Pediatrics..

[35] Dieu O, Llena C, Joing I, Porrovecchio A, Potdevin F (2020). Fun to engage or engage to have fun? Study of different teaching formats in Physical Education. J Phys Educ Sport.

[36] Varea V, González-Calvo G, García-Monge A (2020). Exploring the changes of physical education in the age of Covid-19. Phys Educ Sport Pedagog.

[37] Nettlefold L, McKay HA, Warburton DE, McGuire KA, Bredin SS, Naylor PJ (2011). The challenge of low physical activity during the school day: at recess, lunch and in physical education. Br J Sports Med.

[38] Weaver GR, Tassitano RM, Tenorio MCM, Brazendale K, Beets MW (2021). Temporal trends in Children's school day moderate to vigorous physical activity: a systematic review and Meta-regression analysis. J Phys Act Health.

[39] Bailey R (2006). Physical education and sport in schools: a review of benefits and outcomes. J Sch Health.

[40] Hynynen ST, van Stralen MM, Sniehotta FF (2016). A systematic review of school-based interventions targeting physical activity and sedentary behaviour among older adolescents. Int Rev Sport Exerc Psychol.

[41] Pate RR, Davis MG, Robinson TN (2006). Promoting physical activity in children and youth: a leadership role for schools: a scientific statement from the American Heart Association Council on nutrition, physical activity, and metabolism (physical activity committee) in collaboration with the councils on cardiovascular disease in the young and cardiovascular nursing. Circulation..

[42] Brooke HL, Corder K, Atkin AJ, van Sluijs EM (2014). A systematic literature review with meta-analyses of within- and between-day differences in objectively measured physical activity in school-aged children. Sports Med.

[43] Booth VM, Rowlands AV, Dollman J (2015). Physical activity temporal trends among children and adolescents. J Sci Med Sport.

[44] Tcymbal A, Demetriou Y, Kelso A, Wolbring L, Wunsch K, Wäsche H, Woll A, Reimers AK (2020). Effects of the built environment on physical activity: a systematic review of longitudinal studies taking sex/gender into account. Environ Health Prev Med.

[45] Panter J, Guell C, Humphreys D, Ogilvie D (2019). Title: can changing the physical environment promote walking and cycling? A systematic review of what works and how. Health Place.

[46] Suga AC, Silva AA, Brey JR (2021). Effects of interventions for promoting physical activity during recess in elementary schools: a systematic review. J Pediatr.

[47] Chandler JL, Brazendale K, Drenowatz C, Moore JB, Sui X, Weaver RG, Beets MW (2019). Structure of physical activity opportunities contribution to Children’s physical activity levels in after-school programs. J Phys Act Health.

[48] Müller J (2018). Les jeunes aiment le sport...de préférence sans contraintes.

[49] Belton S, O’Brien W, Issartel J, McGrane B, Powell D (2016). Where does the time go? Patterns of physical activity in adolescent youth.

[50] Scheerder J, Vanreusel B, Taks M, Renson R (2005). Social stratification patterns in adolescents’ active sports participation behaviour: a time trend analysis 1969-1999 ?. Eur Phys Educ Rev.

[51] Laakso L, Telama R, Nupponen H, Rimpela A, Lasse P (2008). Trends in leisure time physical activity among young people in Finland, 1977—2007. Eur Phys Educ Rev.

[52] Vilhjalmsson R, Kristjansdottir G (2003). Gender differences in physical activity in older children and adolescents: the central role of organized sport. Soc Sci Med.

[53] Pate RR, Mitchell JA, Byun W, Dowda M (2011). Sedentary behaviour in youth. Br J Sports Med.

[54] Stappers NEH, Van Kan DHH, De Vries NK, Kremers SPJ (2018). Do physical activity friendly neighborhoods affect community members equally? A cross-sectional study. Int J Environ Res Public Health.

[55] Remmers T, Van Kann D, Kremers S (2020). Investigating longitudinal context-specific physical activity patterns in transition from primary to secondary school using accelerometers, GPS, and GIS. Int J Behav Nutr Phys Act.

[56] WHO guidelines on physical activity and sedentary behaviour. 2020. URL: https://www.who.int/publications/i/item/9789240015128. 33369898

[57] Edwards LC, Bryant AS, Keegan RJ, Morgan K, Jones AM (2017). Definitions, foundations and associations of physical literacy: a systematic review. Sports Med.

[58] Physical Literacy For Life 2021. https://physical-literacy.isca.org.

[59] International Physical Literacy Association. 2017. IPLA definition Retrieved from https://www.physical-literacy.org.uk/

[60] Gulley T (2013). Time perspective and physical activity among central Appalachian adolescents. J Sch Nurs.

[61] Steene-Johannessen J, Anderssen SA, Kolle E, Hansen BH, Bratteteig M, Dalhaug EM, et al. Temporal trends in physical activity levels across more than a decade – a national physical activity surveillance system among Norwegian children and adolescents. Int J Behav Nutr Phys Act. 2021;18-55. 10.1186/s12966-021-01120-z. 10.1186/s12966-021-01120-z PMC8074468 33902618

[62] Virgara R, Phillips A, Lewis L, Richardson M, Maher C. Development of Australian physical activity and screen time guidelines for outside school hours care: an international Delphi study. Int J Behav Nutr Phys Act. 2021;18(3). 10.1186/s12966-020-01061-z. 10.1186/s12966-020-01061-z PMC7789289 33407628

[63] Davids K, Araújo D, Brymer E (2016). Designing affordances for health-enhancing physical activity and exercise in sedentary individuals. Sports Med.

[64] Olds T, Ferrar KE, Gomersall SR, Maher C, Walters JL (2012). The elasticity of time: associations between physical activity and use of time in adolescents. Health Educ Behav.

[65] Ellegård K, Svedin U (2012). Torsten Hägerstrand’s time-geography as the cradle of the activity approach in transport geography. J Transp Geogr.

